# Reviews of attitude research in implementation science require comprehensiveness, accuracy, and specificity

**DOI:** 10.1186/s13012-022-01198-4

**Published:** 2022-05-10

**Authors:** Gregory A. Aarons

**Affiliations:** 1 Department of Psychiatry, University of California San Diego, 9500 Gilman Drive (0812), La Jolla, CA 92093-0812 USA; 2 UC San Diego Altman Clinical and Translational Research Institute-Dissemination and Implementation Science Center (UC San Diego ACTRI DISC), 9500 Gilman Drive (0990), La Jolla, CA 92093-0990 USA; 3 Child and Adolescent Services Research Center, 3665 Kearny Villa Rd., Suite 200N, San Diego, CA 92123 USA

Dear Editors-in-Chief, *Implementation Science*:

I write to voice concerns regarding the article by Fishman, Yang, and Mandell (2021, Vol 16, No. 87) recently published in *Implementation Science*. The concerns include attributions, interpretation of the meaning of measures, and an overly narrow consideration of the extant literature on attitudes in implementation research.

The first concern is attribution regarding the 15-item Evidence-Based Practice Attitude Scale (EBPAS) [1]. The authors state that “The EBPAS developers acknowledge that the EBPAS assesses other constructs, such as knowledge” (pg. 6), but they proceed to cite a paper not by the EBPAS developer [2] for support. The cited paper used the expanded EBPAS-50 [3] that was subsequently made more pragmatic in the EBPAS-36, [4] both of which assess eight additional dimensions of attitudes toward evidence-based practices (EBPs). The EBPAS assesses attitudes, not knowledge. The authors incorrectly attributed their assertion to the EBPAS developer (i.e., this letter’s author). If the attribution were true, appropriate evidence should be clearly documented.

Second, the authors offer an example of an EBPAS item that they assert assesses “knowledge,” but this is incorrect. They give the example of attitudes toward implementation of EBP given requirements to do so by supervisors, an organization, or a state (e.g., policy level) (i.e., EBPAS Requirements subscale). These items were intended to, and do, assess attitudes conditional on the source of directives to use EBP in work with patients and clients. This is a critical issue in implementation as directives to adopt and use new practices often come from those who manage, supervise, or set policy for direct services. This is part of the complex outer and inner contexts of implementation represented in frameworks that address such issues [5, 6]. The EBPAS Requirements subscale squarely focuses on attitudes conditional on the nuances of clinicians’ work environments in practice. Fishman et al.’s claim suggests a misunderstanding or misinterpretation of the EBPAS and a potential misconstrual of differences between assessment of attitudes and knowledge.

Third, the authors state that “EBPAS items refer only to vaguely defined behavioral goals, such as adoption of new practices” (pg. 6). Again, the authors misinterpret the EBPAS, as this widely used instrument was designed to assess attitudes relevant to EBP implementation [1, 7]. The original 15-item EBPAS focuses on EBPs for three subscales, and more general attitudes of openness to new practices in one subscale (Openness subscale). The EBPAS is easily adapted for attitudes toward specific innovations in health care such as attitudes toward cognitive behavioral therapy, measurement-based care, or medication-assisted treatment, and behavioral targets such as EBP use or fidelity. Fishman et al.’s conclusions suggest a cursory evaluation of the EBPAS and ignore the breadth of empirical work conducted on attitudes using the EBPAS and its application since its publication in 2004. For example, an informal search on Google Scholar using the search term “evidence-based practice attitude scale” returned more than 1,500 records. However, the Fishman et al review included no direct references for the original EBPAS or other relevant literature that invoked or used the EBPAS.

Fourth, the authors neglect to include both older and newer studies pertinent to their stated goals in this review. While the exclusion of older studies was noted as a limitation, the exclusion of newer studies was also a limitation. The decision to exclude articles not covered in the Lewis et al review [8] (studies from 1990 to 2018) resulted in a narrow and unrepresentative sample of attitude studies and missed relevant studies including more recent work. If the goal was to focus on attitudes and causal models, then that is the literature that should have been systematically searched and reviewed. Relying on a tangentially related review lacking relevant studies, and not specifically targeting attitudes led to the omission of relevant studies squarely related to the Fishman and colleagues’ own declared issues of interest (i.e., attitudes and causal models). For example, Fig. 1 illustrates a more recent study (i.e., 2020) of attitudes as part of a multilevel causal model examining leadership and attitudes in relation to implementation success [9].

**Fig. 1:**
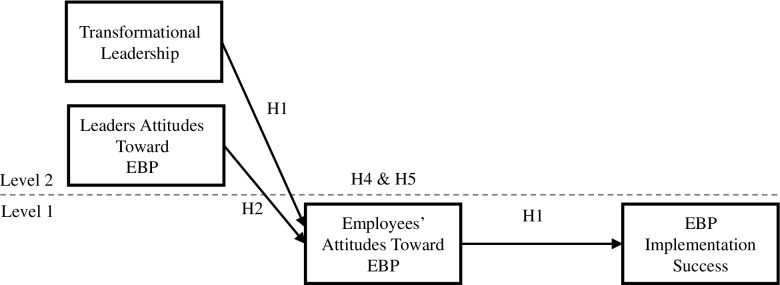
Hypothesized multilevel model of the simultaneous effects of transformational leadership and leaders’ attitudes toward evidence-based practice (EBP) on EBP implementation practice success, as mediated by employees’ attitudes toward EBP

Attitudes operate across multiple phases in complex implementation processes and contexts [5]. It is important for researchers to define key terms and constructs as well as their theoretical origins and underpinnings. Clearly specifying the role of attitudes in the causal chain relating to implementation outcomes is important. While theories may place attitudes in particular causal sequences, attitudes could serve as determinants, mechanisms, or outcomes in causal relationships of interest and can inform theory development and testing of implementation theories and implementation strategies where inferences can be drawn for specified issues of interest using appropriate theoretical models and relevant study designs [10]. For example, Azjen [11] notes that “feedback effects” (where the experience of a behavior can influence subsequent attitudes) is a frequently raised issue in research using the Theory of Planned Behavior. Empirical evidence for “reverse-causal relations” in the Theory of Planned Behavior suggests that reciprocal causal relations should be considered in attitude focused implementation research [12]. Fishman and colleagues appeared to downplay the importance of research on issues such as the role of attitudes related to policies on subsequent intentions and health behaviors (p. 3). However, recent research demonstrates the relevance of the roles of policy, attitudes, and intentions during the COVID-19 health crisis [13]. Thus, causal theory should be clearly specified while remaining open to alternative hypotheses and empirical testing.

Reviews in the field of implementation science should have accurate attributions and should be rigorous and comprehensive to avoid selection bias and misinterpretations, as is consistent with rigor advocated by respected bodies such as Cochrane Reviews [14]. It is not only risk of bias in individual studies, but risk of bias in reviews that can compromise the scientific endeavor [15]. Reviews must be comprehensive regarding the question(s) being asked, identification and use of relevant literature, and employing rigorous methods to avoid formulating conclusions that may not reflect the extant literature. By employing rigorous approaches to reviews, together we can advance the field of implementation science with the highest degree of rigor and relevance.

## Data Availability

Not applicable

## References

[1] Aarons GA (2004). Mental health provider attitudes toward adoption of evidence-based practice: the Evidence-Based Practice Attitude Scale (EBPAS). Ment Health Serv Res.

[2] Edmunds JM, Read KL, Ringle VA, Brodman DM, Kendall PC, Beidas RS (2014). Sustaining clinician penetration, attitudes and knowledge in cognitive-behavioral therapy for youth anxiety. Implement Sci.

[3] Aarons GA, Cafri G, Lugo L, Sawitzky A (2012). Expanding the domains of attitudes towards evidence-based practice: The Evidence-based Practice Attitude Scale-50. Adm Policy Ment Hlth.

[4] Rye M, Torres EM, Friborg O, Skre I, Aarons GA. The Evidence-based Practice Attitude Scale-36 (EPBAS-36): a brief and pragmatic measure of attitudes to evidence-based practice validated in Norwegian and U.S. samples. Implement Sci. 2017;12:44. 10.1186/s13012-017-0573-0 PMC5379724 28372587

[5] Aarons GA, Hurlburt M, Horwitz SM (2011). Advancing a conceptual model of evidence-based practice implementation in public service sectors. Adm Policy Ment Hlth.

[6] Moullin JC, Dickson KS, Stadnick NA, Rabin B, Aarons GA (2019). Systematic review of the Exploration, Preparation, Implementation, Sustainment (EPIS) framework. Implement Sci.

[7] Aarons GA, Glisson C, Hoagwood K, Kelleher K, Landsverk J, Cafri G (2010). Psychometric properties and United States national norms of the Evidence-Based Practice Attitude Scale (EBPAS). Psychol Assessment.

[8] Lewis CC, Boyd MR, Walsh-Bailey C, Lyon AR, Beidas RS, Mittman B, Aarons GA, Weiner BJ, Chambers DA (2020). A systematic review of empirical studies examining mechanisms of implementation in health. Implement Sci.

[9] Farahnak LR, Ehrhart MG, Torres EM, Aarons GA (2020). The influence of transformational leadership and leader attitudes on subordinate attitudes and implementation success. J Leadersh Organ Stud.

[10] Pearl J (1996). Structural and probalistic causality. Psychol Learn.

[11] Ajzen I (2020). The theory of planned behavior: Frequently asked questions. Hum Behav Emerg Technol.

[12] Sussman R, Gifford R (2019). Causality in the theory of planned behavior. Pers Soc Psychol Bull.

[13] Fischer R, Karl JA. Predicting Behavioral Intentions to Prevent or Mitigate COVID-19: A Cross-Cultural Meta-Analysis of Attitudes, Norms, and Perceived Behavioral Control Effects. Social Psychological and Personality. Science. 2021. 10.1177/19485506211019844.

[14] Cipriani A, Furukawa T, Barbui C (2011). What is a Cochrane review?. Epidemiol Psychiatr Sci.

[15] Babic A, Vuka I, Saric F, Proloscic I, Slapnicar E, Cavar J, Pericic TP, Pieper D, Puljak L (2020). Overall bias methods and their use in sensitivity analysis of Cochrane reviews were not consistent. J Clin Epidemiol.

